# Supplementary material for: The role of foreign capital and economic freedom in sustainable food production: Evidence from DLD countries
Source: PLoS One. 2021 Jul 26;16(7):e0255186. doi: 10.1371/journal.pone.0255186 (PMC8312926; doi:10.1371/journal.pone.0255186)
Supplement: S1 Appendix — (DOCX) [file pone.0255186.s001.docx]

# S1 Appendix

**List of Countries sampled for the study (Authors’ own estimations)**

| Developing Countries |  | Least-Developed Countries |  |
| --- | --- | --- | --- |
| Bolivia | Peru | Angola | Laos |
| Botswana | Philippines | Bangladesh | Lesotho |
| Cameroon | Saint Lucia | Benin | Madagascar |
| Cape Verde | Samoa | Burkina Faso | Malawi |
| Dominican Republic | Sri Lanka | Burundi | Mali |
| Ecuador | Suriname | Cambodia | Mauritania |
| El Salvador | Swaziland | The central African Republic | Nepal |
| Fiji | Tajikistan | Chad | Nicaragua |
| Gabon | Tunisia | Congo | Niger |
| Guatemala | Venezuela | Cote d'Ivoire | Nigeria |
| Guyana | Vietnam | Democratic Republic of Congo | Pakistan |
| India |  | Ethiopia | Rwanda |
| Indonesia |  | The Gambia | Senegal |
| Jamaica |  | Ghana | Sierra Leone |
| Mauritius |  | Guinea | Sudan |
| Mongolia |  | Guinea-Bissau | Tanzania |
| Morocco |  | Haiti | Togo |
| Namibia |  | Honduras | Uganda |
| Papua New Guinea |  | Iran | Zambia |
| Paraguay |  | Kenya | Zimbabwe |
