# Supplementary material for: The role of foreign capital and economic freedom in sustainable food production: Evidence from DLD countries
Source: PLoS One. 2021 Jul 26;16(7):e0255186. doi: 10.1371/journal.pone.0255186 (PMC8312926; doi:10.1371/journal.pone.0255186)
Supplement: Attachment — Submitted filename: RESPONSE TO EDITOR AND REVIEWERS.pdf [file pone.0255186.s002.pdf]

Jiangsu University

No 301 Xuefu Road

Zhenjiang – Jiangsu Province

P. R. China

25/06/2021

THE EDITOR

PLOS ONE JOURNAL

Dear Editor,

<u>RESPONSE TO EDITOR/REVIEWERS – PONE-D-21-10241</u>

With reference to your decision to our manuscript submitted with the above-mentioned number, we have done necessary revision. Specifically, the point-by-point revision responses have been outlined below and the responses are in block letters, bold, and italics.

<u>Editor</u>

1. Please ensure that your manuscript meets PLOS ONE's style requirements, including those file naming. ***DONE***
2. We note you have included a table to which you do not refer in the text of your manuscript. ***ALL TABLES AND FIGURE HAVE BEEN CITED IN THE TEXT OF THE MANUSCRIPT.***

<u>Reviewer 1</u>

1. However, I here found the only discrepancy in the paper actually: in the third part of the literature review, writing about economic freedom, we should approach the different economic freedom indexes, at least the famous ones. So I recommend referring it and some works that are using this index for investigating the same field? ***OTHER STUDIES THAT USED THE ECONOMIC FREEDOM INDEXES HAVE BEEN DISCUSSED IN THE LITERATURE REVIEW PART. MOREOVER, TO THE BEST OF OUR KNOWLEDGE, NO STUDY HAS BEEN CONDUCTED CONSIDERING ECONOMIC FREEDOM WITH FOREIGN CAPITAL AND FOOD PRODUCTION – AND IN PARTICULAR USING THE ECONOMIC FREEDOM INDEX.***

Reviewer Two

1. What I see as problem is that I only see the range of variables included and not the data. Thus the research does not meet the criterion of being replicable by anyone. ***THE DATA FOR THE STUDY HAS DEPOSITED AT MENDELEYDATA WITH A DOI- http://dx.doi.org/10.17632/fr2fcvftpj.1.***

Upon carefully studying the reviewers' comments and the highlighted areas for improvement, we can confidently say that we have done all of the recommendations. Moreover, we have included the highlighted portion of the previous manuscript as in the areas revised. Please find this revision and kindly consider our manuscript for publication in your reputable journal. Counting on your usual cooperation. Thank you in anticipation.

Yours Sincerely,

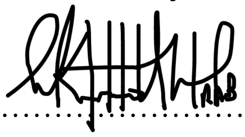

………………………………

Prince Asare Vitenu-Sackey

(Corresponding Author)
